# Variation and Molecular Basis for Enhancement of Receptor Binding of H9N2 Avian Influenza Viruses in China Isolates

**DOI:** 10.3389/fmicb.2020.602124

**Published:** 2020-12-17

**Authors:** Yang Liu, Shuo Li, Huapeng Sun, Liangqi Pan, Xinxin Cui, Xuhui Zhu, Yaling Feng, Mingliang Li, Yanan Yu, Meihua Wu, Jiate Lin, Fengxiang Xu, Shaohua Yuan, Shujian Huang, Hailiang Sun, Ming Liao

**Affiliations:** ^1^College of Veterinary Medicine, South China Agricultural University, Guangzhou, China; ^2^National and Regional Joint Engineering Laboratory for Medicament of Zoonosis Prevention and Control, College of Veterinary Medicine, South China Agricultural University, Guangzhou, China; ^3^Guangdong Laboratory for Lingnan Modern Agriculture, Guangzhou, China; ^4^Key Laboratory of Zoonoses Control and Prevention of Guangdong, Guangzhou, China; ^5^School of Life Science and Engineering, Foshan University, Foshan, China

**Keywords:** influenza, HA, H9N2 AIV, receptors binding site, variation

## Abstract

Currently, H9N2 avian influenza viruses (H9N2 AIVs) globally circulate in poultry and have acquired some adaptation to mammals. However, it is not clear what the molecular basis is for the variation in receptor-binding features of the H9N2 AIVs. The receptor-binding features of 92 H9N2 AIVs prevalent in China during 1994–2017 were characterized through solid-phase ELISA assay and reverse genetics. H9N2 AIVs that circulated in this period mostly belonged to clade h9.4.2. Two increasing incidents occurred in the ability of H9N2 AIVs to bind to avian-like receptors in 2002–2005 and 2011–2014. Two increasing incidents occurred in the strength of H9N2 AIVs to bind to human-like receptors in 2002–2005 and 2011–2017. We found that Q227M, D145G/N, S119R, and R246K mutations can significantly increase H9N2 AIVs to bind to both avian- and human-like receptors. A160D/N, Q156R, T205A, Q226L, V245I, V216L, D208E, T212I, R172Q, and S175N mutations can significantly enhance the strength of H9N2 AIVs to bind to human-like receptors. Our study also identified mutations T205A, D208E, V216L, Q226L, and V245I as the key sites leading to enhanced receptor binding of H9N2 AIVs during 2002–2005 and mutations S119R, D145G, Q156R, A160D, T212I, Q227M, and R246K as the key sites leading to enhanced receptor binding of H9N2 AIVs during 2011–2017. These findings further illustrate the receptor-binding characteristics of avian influenza viruses, which can be a potential threat to public health.

## Introduction

H9N2 avian influenza viruses (AIVs) have low pathogenicity and circulate throughout Asia, the Middle East, and North Africa (Peacock et al., 2019). H9N2 AIVs are among the three major AIV subtypes that affect the poultry industry, causing great economic losses to the poultry industry in China (Gu et al., 2017). In addition to circulating in poultry, H9N2 AIVs have broken through the host barrier and caused infections in many mammals, such as pigs, minks, dogs, and humans (Cong et al., 2007; Su et al., 2014; Peng et al., 2015; Pan et al., 2018).

The first case of human infection with H9N2 AIV was reported in 2000 (Peiris et al., 1999; Butt et al., 2005), but human infection cases have increased in recent years (Pan et al., 2018). A serological survey revealed that 15% of poultry workers had obtained serum against H9N2 AIVs (Huang et al., 2013; Yu et al., 2013). H9N2 viruses with enhanced ability to bind to human receptors are an increasing threat to public health.

The H9N2 viruses can be classified into two distinct lineages based on the hemagglutinin (HA) gene: the North American lineage and the Eurasian lineage. The Eurasian lineage is further divided into three major sublineages: h9.3, h9.4.1, and h9.4.2. The h9.3 lineage was mostly found in chickens and wild birds in Korea, while the h9.4.1 lineage mainly circulates in North Africa, the Middle East, and Southern China. The h9.4.2 lineage is prevalent in Vietnam and China (Guo et al., 2000; Kim et al., 2006, 2013). The h9.4.2 lineage was first isolated in 1994 and became the predominant lineage in China. The h9.4.2 lineage can further spitted into six phylogenetic sublineage referred as h9.4.2.1–h9.4.2.6. The domestic H9N2 viruses isolated before 2007 generally belonged to clades h9.4.2.1 to h9.4.2.4 in chronological order. Hence, during 2007–2013, clades h9.4.2.4, h9.4.2.5, and h9.4.2.6 have co-circulated in China, but h9.4.2.5 viruses are more common than h9.4.2.4 and h9.4.2.6 (Jiang et al., 2012). The clade h9.4.2.6 mainly circulated in Southern China from about 2010. The most prevalent clade of H9N2 viruses in China is clade h9.4.2.5, which was first isolated around 2005 and became pandemic after 2013. Nowadays, H9N2 viruses in the h9.4.1 lineage show a preference for human receptors and enhanced virulence and transmission in mammals (Li et al., 2014; Peacock et al., 2017b). As the major lineage circulating in China, h9.4.2 viruses also have obtained human receptor-binding ability and effective transmissibility in pigs (Sun et al., 2019; Zou et al., 2019).

Some substitutions of amino acids in or near the receptor-binding domain (RBD) enable influenza viruses to acquire the ability to bind to human receptors. The substitutions E190D and G225D for the H1 subtype and substitutions Q226L and G228S for the H2 and H3 subtypes enhance the viruses’ ability to bind α-2,6SA receptors (Matrosovich et al., 2000; Tumpey et al., 2007). The mutations Q226L and G228S increase the ability of H5 and H7 subtypes to bind to human receptors (Maines et al., 2011; Imai and Kawaoka, 2012; Tharakaraman et al., 2013). Besides, mutations N186K, K193R, Q196R, and S227N for the H5 subtype and K193R, G186E, R205G, and S227T for the H7 subtype are associated with increased ability to bind to human receptors (Yamada et al., 2006; Yang et al., 2010; Chen et al., 2012; Gambaryan et al., 2012; Herfst et al., 2012).

The deletion of the 220-loop can also facilitate the binding of H7 and H9 subtypes to human receptors (Yang et al., 2010; Peacock et al., 2017a). T189A, A190V, I155T, G192R, Q226L, and G228S mutations are involved in the enhanced ability of the H9 subtype to bind to human receptors and promote adaptation to infecting mammals such as mice and ferrets (Ha et al., 2001; Sorrell et al., 2009; Li et al., 2014; Teng et al., 2016). Mutation Q226L is no longer considered a molecular marker of the acquisition of human receptor-binding ability for the H9 subtype because of some viruses with 226L exhibiting poor ability to bind to human receptors (Peacock et al., 2017b).

H9N2 viruses are circulating globally, and their threat to public health is increasing. However, the receptor-binding preference is not well characterized, and the molecular basis for the variation in their receptor-binding ability is still unclear. Thus, our study’s purposes were (1) to characterize the receptor-binding variability of H9N2 viruses circulating in China during 1994–2017 and (2) to illustrate the molecular basis for current H9N2 viruses increasing receptor-binding ability.

## Materials and Methods

### Viral Propagation and Identification

H9N2 viruses were isolated from oropharyngeal and cloacal swabs collected from live poultry markets (LPMs) in 1994–2017 from nine provinces (Guangdong, Liaoning, Jiangsu, Shandong, Guangxi, Shanxi, Hainan, Henan, and Fujian province) in China. Viruses were purified using limiting dilution three times in specific pathogen-free (SPF) embryo eggs. Then, HI, NI, and sequencing of HA/NA genes were used for identification and stored in our lab. Viruses were propagated in 9–11-day SPF embryo eggs at 37°C. After 72 h post-inoculation, HA assay was performed using 1% turkey red blood cells (TRBCs). Total RNA was extracted by an RNAfast2000 purification kit (Fastagen Biotech, Shanghai, China) according to the manufacturer’s instructions. Reverse transcription polymerase chain reaction (RT-PCR) was conducted using the Uni12 primer (AGCAAAAGCAGG). The matrix protein (M) gene was amplified using specific primers (Hoffmann et al., 2001), and PCR products were confirmed by agarose gel electrophoresis. The PCR-positive samples were subtyped by hemagglutination inhibition and neuraminidase inhibition assay with H9N2 virus antiserum (Harwc, Harbin, China). The H9N2 viruses were stored in a freezer at −80°C.

### HA Gene Sequencing and Phylogenetic Analysis

Hemagglutinin genes of all H9N2 viruses were amplified by specific primers (Hoffmann et al., 2001). After electrophoresis in 1% agarose gel, PCR products were purified with a Gel Extraction Kit D2500 (Omega Bio-Tek, Guangzhou, China) and sent to Shanghai Invitrogen Biotechnology Co. for sequencing. Sequences were compiled with the SeqMan program of Lasergene 7. A phylogenetic tree of HA genes of H9N2 influenza viruses was generated by the Maximum Likelihood (ML) method with the MEGA 7 software (Sinauer Associates, Inc., Sunderland, MA, United States). Test of phylogeny was made using the bootstrap method with 1,000 bootstrap replicates. Substitution type was nucleotide. The model/method used was the Tamura–Nei model. Rates and sites were set as uniform rates. The gaps/missing data treatment was complete deletion. ML heuristic method was the nearest neighbor interchange (NNI).

### Viral Receptor-Binding Ability Detection by Solid-Phase Direct Binding ELISA

Each virus’ receptor-binding ability was detected by a solid-phase direct binding ELISA assay, as described previously (Sun et al., 2019). Two different synthetic sialylglycopolymers were used: α2,3-linked SA (Neu5Acα2-3Galβ1-4GlcNAcβ-PAA-biotin, 3′-SLN) and α2,6-linked SA (Neu5Acα2-6Galβ1-4GlcNAcβ-PAA-biotin, 6′-SLN) (GlycoTech, Inc., Gaithersburg, MD, United States). Briefly, 96-well plates (Corning 3590, New York, NY, United States) were coated with 100 μl of streptavidin (PuriMag Biotech, Xiamen, China), 10 μg/ml and 37°C for 18–24 h until dry. After washing with phosphate-buffered saline containing 0.05% Tween-20 (0.05% PBST) three times, receptor analogs 3′-SLN and 6′-SLN were added to the plate at a concentration of 7.8–1,000 ng/ml, and culture was carried out at 4°C for 18 h.

The plate was washed with PBS three times and then blocked with 2% skim milk powder at 4°C for 6 h. After washing with 0.05% PBST three times, viruses were added with a titer of 128 HA units and incubated at 4°C for 12 h. After washing with 0.05% PBST three times, a monoclonal antibody against H9N2 HA (Zoonogene, Beijing, China) was added to the plate at a concentration of 0.4 nmol/ml incubated at 4°C for 5 h. After washing with 0.05% PBST three times, the horseradish peroxidase (HRP)-conjugated mouse immunoglobulin G (IgG) antibody with 100 ng/ml concentration was added and incubated at 4°C for 2 h. After washing with 0.05% PBST three times, incubation was done with 3,3′,5,5′-tetramethylbenzidine (TMB) (Solarbio, Beijing, China) at room temperature for 10 min. Then, the ELISA stop solution (Solarbio, Beijing, China) was added to the plate, and the optical density (OD) values were detected at a density of 450 nm.

### Scoring of Related Receptor-Binding Sites

To evaluate the receptor-binding ability of the first H9N2 virus isolated in China, the OD values of avian and human receptor-binding ability of an H9N2 virus, A/chicken/Guangdong/SS/1994(H9N2) (SS94), were detected by ELISA using two different receptor analogs at a concentration of 1,000 ng/ml. Compared to SS94, viruses with higher OD values for either avian- or human-like receptor analogs were classified as strong receptor-binding viruses. Sixty-seven viruses posing higher avian or human receptor-binding ability than SS94 were selected from the 92 H9N2 viruses above for further analysis. The relative OD value for SS94 was designated as the score of this virus. The mutations at 90–260 amino acid sites (H3 numbering) of H9N2 viruses with higher receptor-binding ability were compared to the amino acid sequence of the HA protein of SS94 and calculated using MEGA 7 software. The mutation score is the mutation frequency of this virus.

### Rescuing Recombinant and Mutant Viruses

A recombinant virus with HA and NA genes from SS94 and internal genes from A/Puerto Rico/8/1934 (H1N1) (PR8) was rescued by a reverse genetics system (Hoffmann et al., 2000). According to the manufacturer’s instructions, a single-site mutation in the HA gene was generated by Mut Express^®^ MultiS Fast Mutagenesis Kit V2 (Vazyme, Nanjing, China). Plasmids were extracted using an Endo-free Plasmid Mini Kit II (Omega Bio-Tek, Guangzhou, China) according to the manufacturer’s instructions. HEK 293T cells and Madin–Darby canine kidney cells (MDCKs) were seeded in a six-well plate at a ratio of 10:1 and cultured at 37°C. When the cell confluence was about 90%, 1 μg of each plasmid was transfected using Lipofectamine^TM^ 3000 Transfection Reagent (Invitrogen, L3000015, Carlsbad, CA, United States) according to the manufacturer’s instructions.

The supernatants were collected at 48 h post-transfection and inoculated into 9–11-day SPF embryo eggs and then cultured at 37°C. After 72 h of infection, the rescued viruses were detected by HA assay using 1% TRBCs. Recombinant and mutant viruses were then confirmed by sequencing. The recombinant virus and mutants were stored at −80°C in a freezer.

### Analysis of Receptor-Binding Ability of Mutants

As described previously, the receptor-binding features of mutants were detected by a solid-phase direct binding ELISA assay. Ninety-six-well plates were coated with 3′- or 6′-SLN at a concentration of 3,000 ng/ml, and a titer of 128 HA units viruses was added. The OD values were detected at a density of 450 nm. Each virus was detected with two separate assays.

### Ethics Statements

This study complied with approved protocols of the Biosafety Committee of South China Agriculture University. The protocol (SCAUADL2018-010) was approved by the Experimental Animal Administration and Ethics Committee of the South China Agricultural University.

### Data Statistics and Analyses

All data in this study are processed and analyzed using the software GraphPad Prism (Version 8.4.3). *t* test was used for statistical analysis. Every ELISA experiment was conducted twice and expressed as mean ± SD.

## Results

### Genetic Characterization of HA of H9N2 Influenza Viruses Isolated in China in 1994–2017

The genetic relationships of the viruses from different years and districts were analyzed by sequencing HA genes of 92 viruses collected in live poultry markets (LPMs) during 1994–2017 from nine provinces (Guangdong, Liaoning, Jiangsu, Shandong, Guangxi, Shanxi, Hainan, Henan, and Fujian province) in China (the accession numbers were MT561774–MT561865; Supplementary Table 1). These viruses’ HA genes shared 79.1–96.8% and 72.9–97.9% similarity with SS94 at the nucleotide level and the amino acid level. HA genes’ cleavage motifs were PSRSSR↓GL, indicating that these viruses had low pathogenicity in chickens.

**FIGURE 1:**
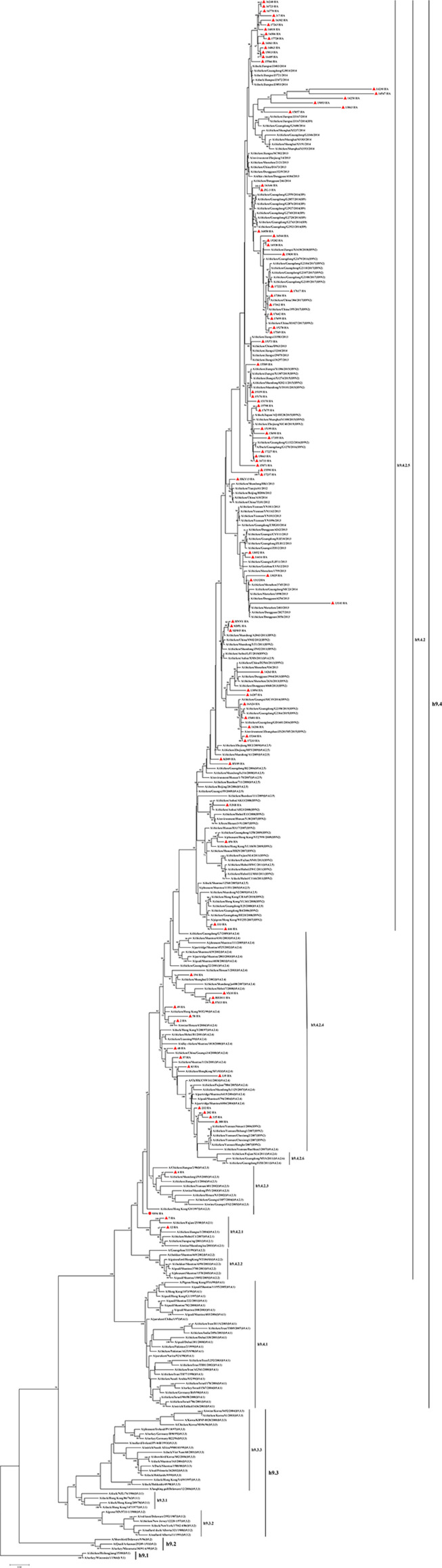
Phylogenetic analysis of the HA gene of 92 H9N2 avian influenza viruses based on nucleotides (nt) 34–1,716. The sequences were aligned by ClustalW. The tree was generated with 1,000 bootstrap replicates by MEGA 7 using a Maximum Likelihood Method. The solid red circle indicates the SS94 virus. Solid red triangles indicate other H9N2 viruses used in this study.

A phylogenetic tree of HA genes of H9N2 viruses was generated by MEGA7 using a Maximum Likelihood method (Figure 1). The results showed that 92 viruses were clustered into 4 clades. Viruses 7 and 12, isolated in 1999 and 2000, belonged to clade h9.4.2.1. Viruses SS94 and 4, isolated in 2000, was in clade h9.4.2.3. Viruses 2, 48, 49, 57, 58, 83, 135, 194, 232, 282, 308, 335, SX10, BD2011, and FX13, isolated in 2000–2013, were in clade h9.4.2.4. The other 74 viruses isolated in 2013–2017 fell into clade h9.4.2.5.

### Receptor-Binding Characteristics of H9N2 Viruses

**FIGURE 2:**
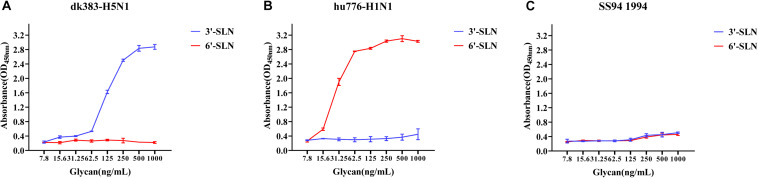
Receptor-binding ability analysis of SS94. **(A)** Receptor-binding ability of dk383-H5N1. **(B)** Receptor-binding ability of hu776-H1N1. **(C)** Receptor-binding ability of SS94. The binding ability of the viruses to two different biotinylated glycans (3′-sialyl-N-acetyllactosamine, 3′-SLN, shown in blue; 6′-sialyl-N-acetyllactosamine, 6′-SLN, shown in red) were detected, and viruses were analyzed at the concentration of 7.8, 15.6, 31.25, 62.5, 125, 250, 500, and 1,000 ng/ml. Every experiment was conducted twice, and the absorbance value was read at 450 nm. The avian H5N1 influenza virus A/Duck/Guangdong/383/2008 (H5N1) (dk383-H5N1) and a human pandemic influenza virus isolate A/Guangdong/776/2015 (H1N1) (hu776-H1N1) were used as typical avian and human tropism control. The H9N2 virus, A/chicken/Guangdong/SS/1994 (SS94), was used as an early stage virus control. The receptor-binding ability of the virus was expressed as mean ± SD.

To illustrate the receptor-binding feature of different clades of H9N2 viruses, the receptor-binding ability of all H9N2 viruses was analyzed by solid-phase direct ELISA, and the results were compared to those of SS94. First, we analyzed those receptor-binding tropism through solid-phase binding assays using an avian H5N1 influenza virus isolate A/Duck/Guangdong/383/2008 (H5N1) (dk383-H5N1) and a human pandemic influenza virus isolate A/Guangdong/776/2015 (H1N1) (hu776-H1N1) as the control that showed typical avian or human receptor-binding tropism (HRBT) (Figures 2A,B). The results showed that SS94 weakly binds to either avian or human receptor analogs (Figure 2C), indicating that H9N2 viruses isolated early showed dual receptor-binding tropism (DRBT). A virus with the higher binding ability to bind to avian receptors than SS94 was considered a strong tropism of avian receptors. Similarly, a virus with the higher binding ability to human receptors than that of SS94 was considered a strong tropism of human receptors. A virus with a lower binding ability to human or avian receptors than SS94 was considered weak tropism.

**FIGURE 3:**
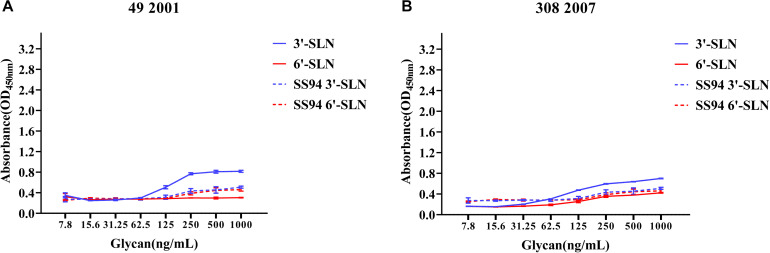
Receptor-binding ability analysis of H9N2 viruses with avian receptor-binding tropism (ARBT). Viruses with stronger avian receptor-binding ability but weaker human receptor-binding ability than the SS94 are classified as ARBT. Receptor binding properties of different H9N2 viruses [indicated as panels **(A,B)**] were tested by solid-phase ELISA. Virus binding ability was measured by two different receptor analogs: 3′-SLN (shown in blue) and 6′-SLN (shown in red). Every experiment was conducted twice and expressed as mean ± SD.

**FIGURE 4:**
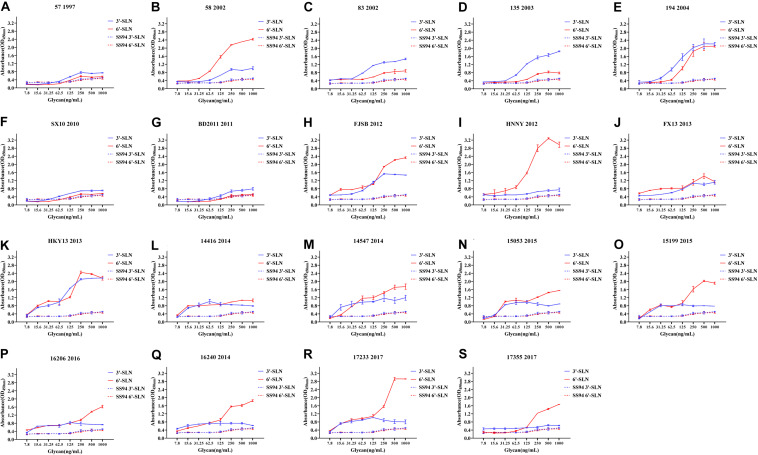
Receptor-binding ability analysis of H9N2 viruses with dual receptor-binding tropism (DRBT). Viruses with the stronger avian receptor-binding ability and human receptor-binding ability than the SS94 are classified as DRBT. Receptor-binding properties of different H9N2 viruses [indicated as panels **(A–S)**] were tested by solid-phase ELISA. Virus binding ability was measured by two different receptor analogs: 3′-SLN (shown in blue) and 6′-SLN (shown in red). Every experiment was conducted twice and expressed as mean ± SD.

**FIGURE 5:**
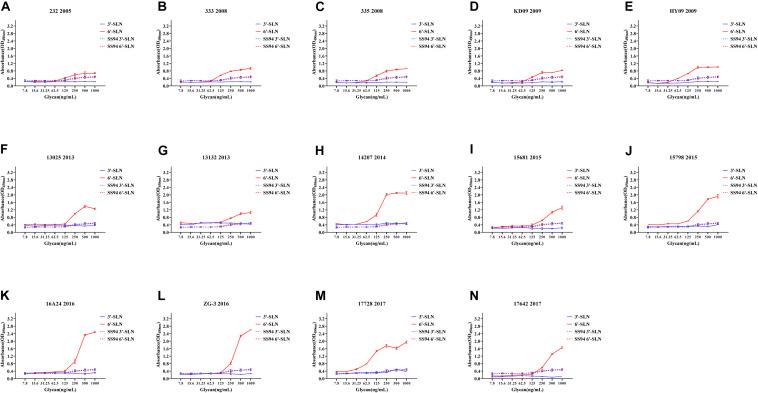
Receptor binding ability analysis of H9N2 viruses with human receptor-binding tropism (HRBT). Viruses with stronger human receptor-binding ability but weaker avian receptor-binding ability than the SS94 are classified as HRBT. Receptor-binding properties of different H9N2 viruses [indicated as panels **(A–N)**] were tested by solid-phase ELISA. Virus binding ability was measured by two different receptor analogs: 3′-SLN (shown in blue) and 6′-SLN (shown in red). Every experiment was conducted twice and expressed as mean ± SD.

**FIGURE 6:**
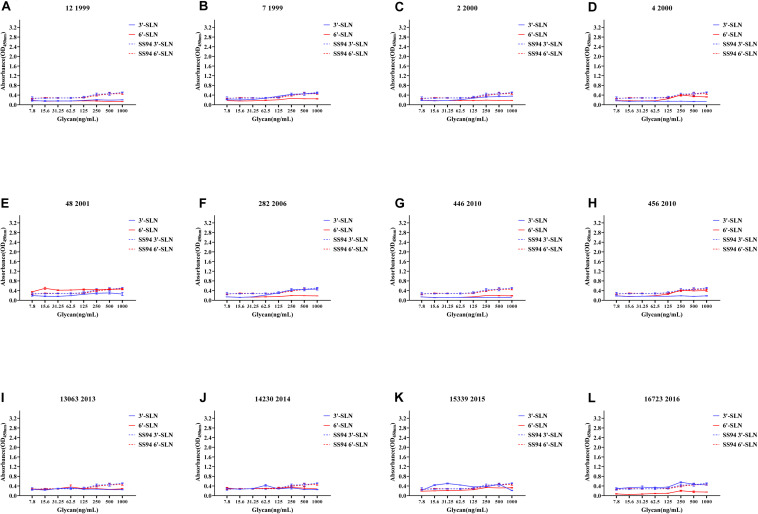
Receptor-binding ability analysis of H9N2 viruses with weakened receptor-binding tropism (WRBT). Viruses with weaker avian and human receptor-binding ability than the SS94 are classified as WRBT. Receptor-binding properties of different H9N2 viruses [indicated as panels **(A–L)**] were tested by ELISA. Virus binding ability was measured by two different receptor analogs: 3′-SLN (shown in blue) and 6′-SLN (shown in red). Every experiment was conducted twice and expressed as mean ± SD.

Viruses with avian and human strong tropisms were classified as having DRBT; those with only one strong tropism were classified as having avian receptor-binding tropism (ARBT) or HRBT. Viruses with two weak tropisms were classified as having weak receptor-binding tropism (WRBT). The ELISA results showed that two viruses exhibited ARBT (Figure 3), 40 viruses exhibited DRBT (Figure 4 and Supplementary Figure 1), 36 viruses showed HRBT (Figure 5 and Supplementary Figure 2), and the other 14 viruses showed WRBT (Figure 6).

The two viruses located in clade h9.4.2.1, strains 7 and 12, showed WRBT. Virus 4 in clade h9.4.2.3 showed WRBT. Among the 15 viruses located in clade h9.4.2.4, three strains showed WRBT, two strains showed ARBT, eight strains showed DRBT, and two strains showed HRBT. The results also indicated that, among 74 viruses in clade h9.4.2.5, there were eight strains with WRBT, 32 strains with DRBT, and 34 strains with HRBT. These findings indicate that, compared to the early stage viruses, most H9N2 viruses have a higher binding ability to avian- and human-like SA receptors, especially for the viruses isolated after 2013, which showed DRBT and HRBT.

**FIGURE 7:**
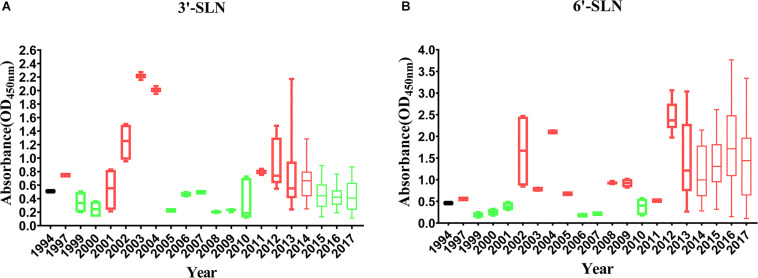
Variation in receptor binding strength of H9N2 viruses from 1994 to 2017: **(A)** Variation in the binding strength of avian receptor (3′-SLN); **(B)** variation in the binding strength of human receptor (6′-SLN). The receptor binding strength is considered to be increased (shown in red) or decreased (shown in green) if its average receptor-binding strength in that year is higher or lower than SS94. A peak is considered to have increased or decreased continuously for more than 3 years. Every virus was conducted twice and expressed as mean ± SD.

To analyze the correlation between the enhancement of receptor-binding ability and isolation year of the virus, the receptor-binding strength of viruses isolated from different years was detected by ELISA with 1,000 ng/ml 3′-SLN/6′-SLN. If the average value was higher than that of SS94, the receptor-binding ability’s strength was considered to increase. If the average value was lower than that of SS94, the strength of the receptor-binding ability was considered to decrease. A serial increase or decrease for more than 3 years was considered a changing peak of viruses’ receptor-binding ability. The results indicated that, in 1994–2017, the binding strength to the avian receptors showed two increasing and three decreasing trends (Figure 7A). Two high peaks enhancing the strength of binding to avian receptors occurred in 2002–2004 and 2011–2014, and three weakening incidents occurred in 1999–2001, 2008–2010, and 2015–2017. After 2015, the strength of binding to avian receptors returned to a level close to that of SS94. The variation from 1994 to 2017 tended to maintain stable strength of binding to avian receptors like early strains. The strength changes of H9N2 viruses to bind to human receptors showed two enhancements and one decreasing trend (Figure 7B). The first enhancement peak appeared in 2002–2005, and the second enhancement event occurred in 2011–2017. A decrease occurred in 1999–2001. The evolutionary tendency of viruses circulated from 1994 to 2017 showed increasing binding strength to human receptors. In 2002–2005 and 2011–2017, the binding ability of H9N2 viruses to either avian or human receptors increased.

### Screening of Amino Acid Sites Involved With the Increasing Ability of H9N2 Viruses to Bind to Receptors

To figure out what mutations are involved with enhancing H9N2 viruses’ ability to bind to avian and human receptors, variations in amino acid sites 90–260 were counted by comparison with SS94. The results showed 19 amino acids involved in increasing H9N2 viruses’ ability to bind to avian- and human-like receptors screened out (Table 1). Of these, 137, 156, 159, 160, 190, 192, 226, and 227 amino acid sites were located in the RBD of viruses, and the other mutations were outside of the RBD. A total of 19 different mutations were selected for further research: M99L, S119R, K141N, K141T, D145N, D145G, Q156R, A160N, A160D, R172Q, S175N, T205A, D208E, T212I, V216L, Q226L, Q227M, V245L, and R246K.

**TABLE 1:** Top 20 mutation sites of H9N2 AIVs with enhancing 3′-SLN/6′-SLN binding strength (H3 numbering).

3′-SLN enhancing prediction sites				6′-SLN enhancing prediction site			
Rank	Amino acid sites	Mutation	Score (Percentage^a^)	Rank	Amino acid sites	Mutation	Score (Percentage^b^)
1	175	S→N	100%	1	175	S→N	100%
2	99	M→L	96.87%	2	99	M→L	98.64%
3	205	T→A	96.87%	3	205	T→A	98.64%
4	216	V→L	96.87%	4	216	V→L	97.29%
5	190	A→T	78.12%	5	208	D→E	89.18%
6	226	Q→L	71.87%	6	226	Q→L	89.18%
7	119	S→R	68.75%	7	190	A→T	86.48%
8	172	R→Q	68.75%	8	245	V→I	86.48%
9	208	D→E	68.75%	9	172	R→Q	85.13%
10	245	V→I	65.62%	10	119	S→R	83.78%
11	227	Q→M	62.5%	11	227	Q→M	81.08%
12	246	R→K	56.25%	12	246	R→K	75.67%
13	145	D→G/N	50.00%	13	145	D→G	68.91%
14	141	K→N/T	46.87%	14	212	T→I	68.91%
15	159	N→G	46.87%	15	248	N→D	67.56%
16	160	A→D	46.87%	16	160	A→D/N	64.86%
17	248	N→D	46.87%	17	141	K→N/T	63.51%
18	137	S→D	43.75%	18	137	S→D	62.16%
19	192	T→R	43.75%	19	156	Q→R/K	60.81%
20	156	Q→R	40.62%	20	159	N→G	60.81%

*^*a*^A total of 32 viruses with enhanced binding strength of 3′-SLN were calculated. ^*b*^A total of 74 viruses with enhanced binding strength of 6′-SLN were calculated.*

### Molecular Basis for the Increasing Ability of H9N2 Viruses to Bind to Receptors

To illustrate the molecular basis for enhancing the ability of H9N2 viruses to bind to receptors, a recombinant virus with HA and NA genes from SS94 and internal genes from A/Puerto Rico/8/1934 (H1N1) (PR8) was rescued by reverse genetics according to a previous study (Hoffmann et al., 2000) and named as rSS94. Then, the following mutations were generated in the HA gene of the recombinant virus: M99L, S119R, K141N, K141T, D145N, D145G, Q156K, A160N, A160D, R172Q, S175N, T205A, D208E, T212I, V216L, Q226L, Q227M, V245L, and R246K. Mutants were rescued and designated as rSS94-M99L, rSS94-S119R, rSS94-K141N, rSS94-K141T, rSS94-D145N, rSS94-D145G, rSS94-Q156R, rSS94-A160N, rSS94-A160D, rSS94-R172Q, rSS94-S175N, rSS94-T205A, rSS94-D208E, rSS94-T212I, rSS94-V216L, rSS94-Q226L, rSS94-Q227M, rSS94-V245I, and rSS94-R246K (Table 2).

**TABLE 2:** Information of recombinant viruses used in this study.

Strain name	Internal genes^a^	HA/NA gene	Mutation (H3 numbering)
rSS94	PR8^b^	SS94^c^	None
rSS94-M99L	PR8	SS94	M99L
rSS94-S119R	PR8	SS94	S119R
rSS94-K141N	PR8	SS94	K141N
rSS94-K141T	PR8	SS94	K141T
rSS94-D145G	PR8	SS94	D145G
rSS94-D145N	PR8	SS94	D145N
rSS94-Q156R	PR8	SS94	Q156R
rSS94-A160D	PR8	SS94	A160D
rSS94-A160N	PR8	SS94	A160N
rSS94-R172Q	PR8	SS94	R172Q
rSS94-S175N	PR8	SS94	S175N
rSS94-T205A	PR8	SS94	T205A
rSS94-D208E	PR8	SS94	D208E
rSS94-T212I	PR8	SS94	T212I
rSS94-V216L	PR8	SS94	V216L
rSS94-Q226L	PR8	SS94	Q226L
rSS94-Q227M	PR8	SS94	Q227M
rSS94-V245I	PR8	SS94	V245I
rSS94-R246K	PR8	SS94	R246K

*^*a*^Internal genes indicate the PB2, PB1, PA, NP, M, and NS gene of influenza virus. ^*b*^PR8 is the abbreviation of the virus A/Puerto Rico/8/1934 (H1N1). ^*c*^SS94 is the abbreviation of the virus A/chicken/Guangdong/SS/1994(H9N2).*

**FIGURE 8:**
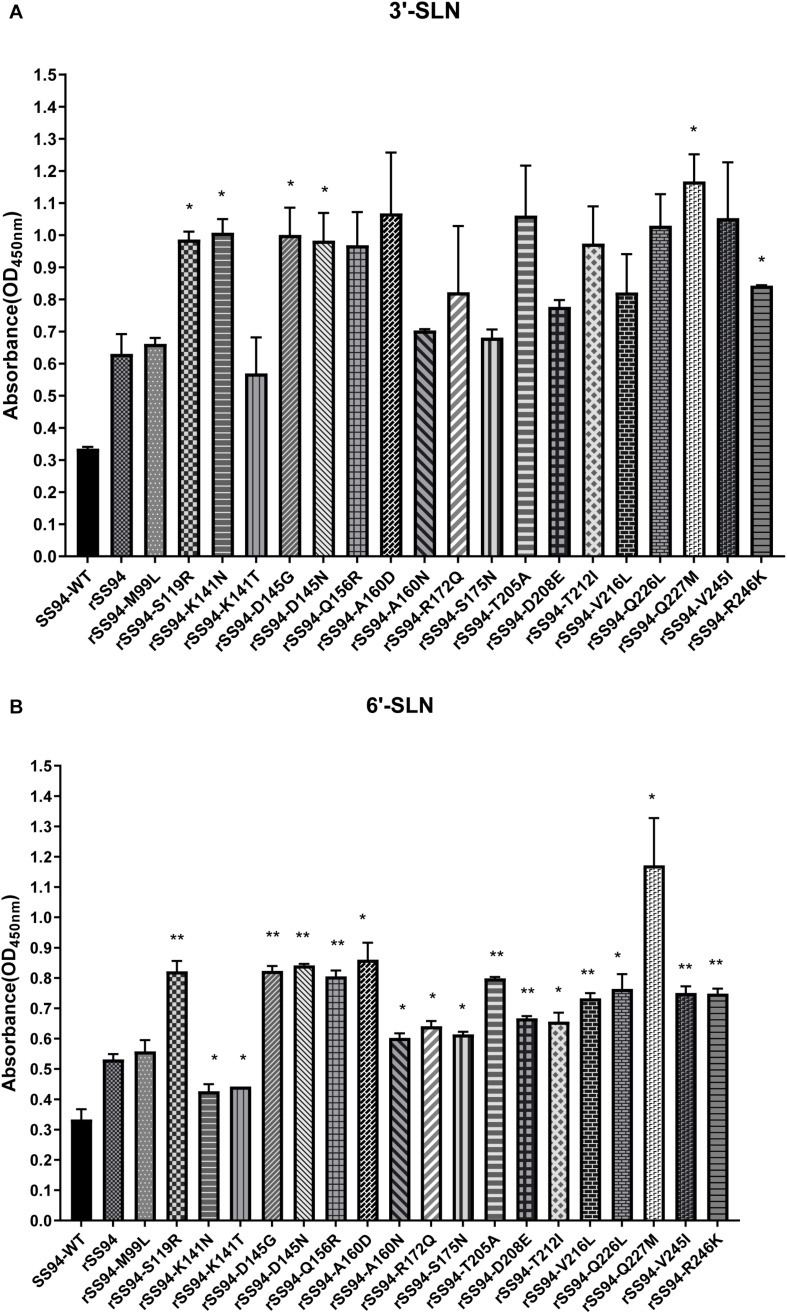
The analysis of receptor-binding ability of H9N2 recombinant viruses with the corresponding mutation in HA protein. **(A)** Receptor-binding strength of avian receptor (3′-SLN); **(B)** Receptor-binding strength of the human receptor (6′-SLN). *t* test was used for statistical analysis. *P < 0.05, **P < 0.01.

The receptor-binding strength of viruses was analyzed using ELISA with 3,000 ng/ml of receptor analogs. The results indicated that rSS94 had the same receptor-binding tropism as SS94 and showed higher receptor-binding strength. Compared to rSS94, the mutants rSS94-S119R, rSS94-K141N, rSS94-D145G, rSS94-D145N, rSS94-Q227M, and rSS94-R246K significantly enhanced the binding strength of 3′-SLN (*P* < 0.05) (Figure 8A). The mutations in order of increasing binding strength of 3′-SLN are as follows: Q227M > K141N > D145G > S119R > D145N > R246K.

The rSS94-K141N and rSS94-K141T mutations caused a significant reduction in the binding strength of 6′-SLN (*P* < 0.05). The other mutants caused a statistical significant increase in the binding strength to 6′-SLN (rSS94-Q227M, rSS94-A160D, rSS94-Q226L, rSS94-T212I, rSS94-R172Q, rSS94-S175N, rSS94-A160N, *P* < 0.05; rSS94-D145N, rSS94-D145G, rSS94-S119R, rSS94-Q156R, rSS94-T205A, rSS94-V245I, rSS94-R246K, rSS94-V216L, rSS94-D208E, *P* < 0.01) (Figure 8B). The ranking was as follows: Q227M > A160D > D145N > D145G > S119R > Q156R > T205A > Q226L > V245I > R246K > V216L > D208E > T212I > R172Q > S175N > A160N.

In summary, mutations Q227M, D145G, D145N, S119R, and R246K can significantly enhance the binding strength of the H9N2 virus to both avian and human receptors. Mutations A160D/N, Q156R, T205A, Q226L, V245I, V216L, D208E, T212I, R172Q, and S175N can significantly enhance the binding strength to human receptors. The mutation K141N can significantly increase the binding strength to avian receptors. The mutation K141T caused a significant reduction in the human receptors (*P* < 0.05).

### Comparative Analysis of Amino Acid Sites Involved With Receptor-Binding Ability Across Natural H9N2 Viruses in China

**FIGURE 9:**
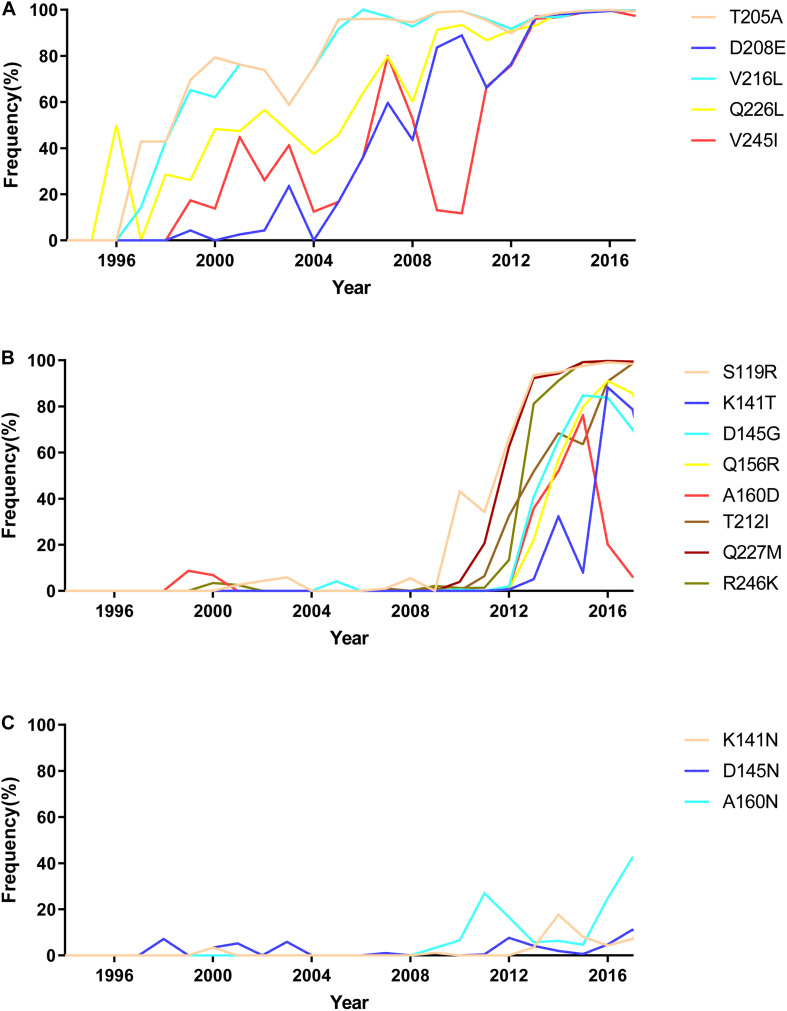
The mutation analysis of HA protein of natural H9N2 viruses circulated in China from 1994 to 2017. **(A)** High-frequency mutations that occurred in natural isolated H9N2 viruses during 2002–2005; **(B)** high-frequency mutations that occurred in natural isolated H9N2 viruses during 2011–2017; **(C)** mutations showed low frequency in natural isolated H9N2 viruses.

To illustrate the receptor-binding feature of H9N2 viruses circulated in 1994–2018 in China, we analyzed the mutations involved with receptor-binding ability of 5,102 HA sequences downloaded from the NCBI Influenza Database (as of September 28, 2019). Our previous results indicated that the receptor-binding ability of H9N2 viruses peaked twice in 1994–2017: once in 2002–2005 and again in 2011–2017. At the first peak, mutations of T205A, V216L, Q226L, V245I, and D208E in HA protein occurred at high frequency (Figure 9A). During this period, mutations Q226L, T205A, V216L, V245I, and D208E played an important role in increasing the binding ability to human receptors.

205A, 208E, 216L, and 226L were gradually stable in the HA protein of H9N2 viruses after 2005. Besides, substitutions S119R, D145G, Q156R, A160D, T212I, Q227M, and R246K in H9N2 viruses rapidly increased after 2012 (Figure 9B). This result indicated that S119R, D145G, Q227M, and R246K were key mutations for H9N2 viruses to maintain DRBT in 2011–2017. The mutations Q156R, A160D, and T212I enhanced the ability of viruses to bind to human-like receptors. Interestingly, a significant increase in the frequency of the mutation of K141T was observed to weaken H9N2 viruses’ ability to bind to human receptors starting in 2013. The results also showed some rare mutations in H9N2 viruses, such as K141N, A160N, and D145N (Figure 9C).

## Discussion

Acquiring the ability to bind to α2,6-linked SA was considered a critical factor for the interspecies transmission of AIVs and adapting to mammals. The receptor-binding ability of the H9N2 viruses in China showed two peaks of increased binding to avian- and human-linked receptors in 2002–2005 and 2011–2017. The currently circulating H9N2 viruses exhibit dual or HRBT. Q226L, V216L, V245I, and D208E are crucial mutations that account for the variation in receptor-binding ability of H9N2 viruses in 2002–2005. Mutations S119R, D145G, Q156R, A160D, T212I, Q227M, and R246K are the molecular basis for changes in the H9N2 receptor-binding ability in 2011–2017.

A minority of H9N2 viruses in the present study fell into clades h9.4.2.1, h9.4.2.3, and h9.4.2.4, and a majority of them belonged to h9.4.2.5. H9N2 viruses have predominantly belonged to the h9.4.2.5 lineage in China since 2013. These findings are consistent with previous studies (Shen et al., 2015; Liu et al., 2016; Zou et al., 2019). No h9.4.2.2 or h9.4.2.6 sublineage viruses were isolated. The clade h9.4.2.2 of H9N2 viruses mainly circulated in a minority of poultry in Guangdong from 1999 to 2007 (Jiang et al., 2012). Previous studies found that the clade h9.4.2.6 of H9N2 viruses have usually been isolated from vaccinated poultry flocks since 2010. This clade had only a limited spread in poultry farms in Southern China and was rarely isolated in LBMs (Chen et al., 2013; Shen et al., 2015; Xu et al., 2018). In this study, samples were mainly collected from chicken in the LBMs, which may explain the possible reason for the lack of clades h9.4.2.2 and h9.4.2.6.

H9N2 viruses currently circulating in China have acquired a strong ability to bind to human receptors since 2013, which is consistent with a previous study (Zou et al., 2019). H9N2 viruses were prevalent in China and underwent tremendous reassortment in 2006–2008. Genotypes A and H disappeared before 2007 (Zhang et al., 2008), multiple genotypes circulated in chickens in 2008–2010, and the S genotype gradually became predominant in China since 2013 (Liu et al., 2016). The results showed that H9N2 viruses had great changes in receptor-binding ability in 2007–2013. The diversity of H9N2 viruses in this period in China may account for changes in receptor-binding ability.

Mutations in Q227P and Q226L were proven to increase both avian and human receptors’ binding ability (Ha et al., 2001; Peacock et al., 2017b). In this study, we also found that mutations Q226L and Q227M can increase the binding ability of both avian and human receptors. Mutations N145K, T156N in H3, T160A in H5, and R205G in H7 subtypes have been found to affect the binding ability of SA (Yang et al., 2010; Huang et al., 2017; Zuo et al., 2018; Santos et al., 2019). In this study, mutations D145G, Q156R, A160D, and T205A were found to increase the receptor-binding ability of H9N2 viruses. The frequency of mutations 119R, 145G, 160D, 212I, 227M, and 246K in H9N2 viruses in China increased rapidly since 2012. These data further prove that the replacements S119R, Q227M, D145G, and A160D were crucial for viruses to enhance human receptor-binding strength in 2012–2017.

Some key mutations in HA protein are not only involved with receptor-binding ability of viruses but also affect the infection and antigenicity of the viruses. Mutations T156N, T212C/E, and N216R/C in H3, as well as T160A and K216E in H5 subtypes, have been found to modulate the pH of membrane fusion (Godley et al., 1992; Lin et al., 1997; Yamada et al., 2006; Keleta et al., 2008; Yang et al., 2010; Dubois et al., 2011; Gambaryan et al., 2012; Herfst et al., 2012). In HA protein, 145, 156, and 160 amino acid sites are considered as antigenic sites of H3 and H5 subtypes of influenza viruses, and mutations that occur at such sites affect the antigenicity of viruses (Abente et al., 2016; Huang et al., 2017; Wang et al., 2017; Zuo et al., 2018; Santos et al., 2019). Whether the mutations involved with the receptor-binding feature of H9N2 viruses in the present study change the other biological characteristics of viruses needs further study.

Our findings indicated that 2005–2011 was a key time for H9N2 viruses to acquire the stronger receptor-binding ability to α2,6-linked SA. During 2005–2011, four human infection cases were reported. Notably, human infection cases of H9N2 viruses have been increasing to 28 during 2012–2019 (Song and Qin, 2020). Besides, it seems that H9N2 viruses currently circulating in poultry can easily transmit between species and infect different mammals. Serological studies indicated an increase in the seropositivity of H9N2 viruses in pig flocks after 2010 (Pusch and Suarez, 2018). Farmed minks have also been reported to be infected with H9N2 viruses since 2013 (Peng et al., 2015; Yongfeng et al., 2017). These findings proved that currently, H9N2, with enhanced ability to bind to α2,6-linked SA, easily break through species barriers to infect mammals, including humans.

Mutations S119R, K141N, D208E, T212I, V216L, V245I, and R246K were first reported in the H9N2 receptor-binding feature in the present study. The change in H9N2 viruses’ receptor-binding ability is associated with multiple amino acid sites in HA protein and other genes. Further studies are urgently needed to fully illustrate the molecular basis for the variation of H9N2 viruses.

## Data Availability Statement

The datasets presented in this study can be found in online repositories. The names of the repository/repositories and accession number(s) can be found in the article/Supplementary Material.

## Ethics Statement

The animal study was reviewed and approved by Experimental Animal Administration and Ethics Committee of the South China Agricultural University.

## Author Contributions

HS designed the experiments, wrote and revised the manuscript. YL conducted the experiments, analyzed the data, and wrote the manuscript. SL, HPS, LP, XC, XZ, YF, ML, YY, MW, and JL conducted the experiments. FX and SY revised the manuscript. ML and SH designed the experiments and revised the manuscript.

## Conflict of Interest

The authors declare that the research was conducted in the absence of any commercial or financial relationships that could be construed as a potential conflict of interest.
