# Supplementary material for: Variation and Molecular Basis for Enhancement of Receptor Binding of H9N2 Avian Influenza Viruses in China Isolates
Source: Front Microbiol. 2020 Dec 17;11:602124. doi: 10.3389/fmicb.2020.602124 (PMC7773702; doi:10.3389/fmicb.2020.602124)
Supplement: Supplementary file 1 [file Table_1.DOCX]

Table S1: List of 92 H9N2 Avian influenza virus strains

| Strain name | Accession number | Abbreviation | Clade | Tropsim | Absorbance (avian/human, 450nm) |
| --- | --- | --- | --- | --- | --- |
| A/Chicken/Guangdong/SS/1994 | AAK62979 | SS94 | h9.4.2.3 | DRBT | 0.509/0.46 |
| A/Chicken/liaoning/57/1997 | MT561851 | 57 | h9.4.2.4 | DRBT | 1.112/0.556 |
| A/Chicken/Jiangsu/12/1999 | MT561774 | 12 | h9.4.2.1 | WRBT | 0.204/0.131 |
| A/Chicken/Shandong/7/1999 | MT561853 | 7 | h9.4.2.1 | WRBT | 0.485/0.245 |
| A/Chicken/Guangdong/2/2000 | MT561839 | 2 | h9.4.2.4 | WRBT | 0.357/0.178 |
| A/Chicken/Shandong/4/2000 | MT561846 | 4 | h9.4.2.3 | WRBT | 0.133/0.322 |
| A/Duck/Guangxi/48/2001 | MT561849 | 48 | h9.4.2.4 | WRBT | 0.258/0.474 |
| A/Chicken/Guangdong/49/2001 | MT561850 | 49 | h9.4.2.4 | ARBT | 0.816/0.305 |
| A/Chicken/Guangdong/83/2002 | MT561854 | 83 | h9.4.2.4 | DRBT | 1.477/0.887 |
| A/Chicken/Shandong/58/2002 | MT561852 | 58 | h9.4.2.4 | DRBT | 1.004/2.44 |
| A/Chicken/Guangdong/135/2003 | MT561780 | 135 | h9.4.2.4 | DRBT | 1.851/0.782 |
| A/Chicken/Guangdong/194/2004 | MT561838 | 194 | h9.4.2.4 | DRBT | 2.215/2.105 |
| A/Chicken/Guangdong/232/2005 | MT561840 | 232 | h9.4.2.4 | HRBT | 0.226/0.677 |
| A/Chicken/Guangdong/282/2006 | MT561841 | 282 | h9.4.2.4 | WRBT | 0.465/0.182 |
| A/Chicken/Guangdong/308/2007 | MT561842 | 308 | h9.4.2.4 | ARBT | 0.701/0.423 |
| A/Chicken/Guangdong/335/2008 | MT561844 | 335 | h9.4.2.4 | HRBT | 0.192/0.918 |
| A/Chicken/Guangdong/333/2008 | MT561843 | 333 | h9.4.2.5 | HRBT | 0.212/0.937 |
| A/Chicken/Shandong/HY09/2009 | MT561860 | HY09 | h9.4.2.5 | HRBT | 0.233/1.01 |
| A/Chicken/Shandong/KD09/2009 | MT561861 | KD09 | h9.4.2.5 | HRBT | 0.221/0.832 |
| A/Chicken/Shanxi/SX10/2010 | MT561864 | SX10 | h9.4.2.4 | DRBT | 0.712/0.566 |
| A/Chicken/Guangdong/446/2010 | MT561847 | 446 | h9.4.2.5 | WRBT | 0.122/0.185 |
| A/Chicken/Hainan/456/2010 | MT561848 | 456 | h9.4.2.5 | WRBT | 0.183/0.402 |
| A/Chicken/Shandong/BD2011/2011 | MT561855 | BD2011 | h9.4.2.4 | DRBT | 0.796/0.518 |
| A/Chicken/Fujian/FJSB/2012 | MT561856 | FJSB | h9.4.2.5 | DRBT | 1.473/2.332 |
| A/Chicken/Shandong/SDPL/2012 | MT561862 | SDPL | h9.4.2.5 | DRBT | 0.579/2.065 |
| A/Chicken/Shandong/SDWF/2012 | MT561863 | SDWF | h9.4.2.5 | DRBT | 0.739/2.385 |
| A/Chicken/Henan/HNNY/2012 | MT561859 | HNNY | h9.4.2.5 | DRBT | 0.748/2.971 |
| A/Chicken/Shandong/FX13/2013 | MT561857 | FX13 | h9.4.2.4 | DRBT | 1.122/1.152 |
| A/Chicken/Shandong/HKY13/2013 | MT561858 | HKY13 | h9.4.2.5 | DRBT | 2.165/2.16 |
| A/Chicken/Guangdong/13025/2013 | MT561775 | 13025 | h9.4.2.5 | HRBT | 0.382/1.256 |
| A/Chicken/Guangdong/13052/2013 | MT561776 | 13052 | h9.4.2.5 | DRBT | 0.596/3.024 |
| A/Chicken/Guangdong/13063/2013 | MT561777 | 13063 | h9.4.2.5 | WRBT | 0.247/0.281 |
| A/Chicken/Guangdong/13094/2013 | MT561778 | 13094 | h9.4.2.5 | DRBT | 0.619/0.623 |
| A/Chicken/Guangdong/13132/2013 | MT561813 | 13132 | h9.4.2.5 | HRBT | 0.438/1.051 |
| A/Partridge/Guangdong/13141/2013 | MT561779 | 13141 | h9.4.2.5 | HRBT | 0.473/2.328 |
| A/Chicken/Guangdong/14207/2014 | MT561781 | 14207 | h9.4.2.5 | HRBT | 0.449/2.096 |
| A/Chicken/Guangdong/14230/2014 | MT561782 | 14230 | h9.4.2.5 | WRBT | 0.255/0.281 |
| A/Chicken/Guangdong/14250/2014 | MT561783 | 14250 | h9.4.2.5 | DRBT | 0.769/0.915 |
| A/Chicken/Guangdong/14263/2014 | MT561784 | 14263 | h9.4.2.5 | DRBT | 0.568/0.623 |
| A/Chicken/Guangdong/14416/2014 | MT56178 | 14416 | h9.4.2.5 | DRBT | 0.79/1.063 |
| A/Chicken/Guangdong/14547/2014 | MT561786 | 14547 | h9.4.2.5 | DRBT | 1.196/1.744 |
| A/Chicken/Guangdong/15053/2015 | MT561787 | 15053 | h9.4.2.5 | DRBT | 0.889/1.552 |
| A/Chicken/Guangdong/15057/2015 | MT561788 | 15057 | h9.4.2.5 | DRBT | 0.666/0.886 |
| A/Chicken/Guangdong/15344/2015 | MT561795 | 15344 | h9.4.2.5 | HRBT | 0.434/0.906 |
| A/Chicken/Guangdong/15282/2015 | MT561793 | 15282 | h9.4.2.5 | DRBT | 0.607/1.311 |
| A/Chicken/Guangdong/15373/2015 | MT561796 | 15373 | h9.4.2.5 | HRBT | 0.363/1.32 |
| A/Chicken/Guangdong/15071/2015 | MT561814 | 15071 | h9.4.2.5 | HRBT | 0.446/0.973 |
| A/Chicken/Guangdong/15566/2015 | MT561797 | 15566 | h9.4.2.5 | DRBT | 0.857/1.715 |
| A/Pigeon/Guangdong/15589/2015 | MT561798 | 15589 | h9.4.2.5 | HRBT | 0.366/0.956 |
| A/Chicken/Guangdong/15590/2015 | MT561799 | 15590 | h9.4.2.5 | HRBT | 0.133/0.722 |
| A/Chicken/Guangdong/15813/2015 | MT561803 | 15813 | h9.4.2.5 | HRBT | 0.246/2.582 |
| A/Environment/Guangdong/15170/2015 | MT561789 | 15170 | h9.4.2.5 | HRBT | 0.451/1.946 |
| A/Chicken/Guangdong/15278/2015 | MT561792 | 15278 | h9.4.2.5 | DRBT | 0.526/0.969 |
| A/Chicken/Guangdong/15339/2015 | MT561794 | 15339 | h9.4.2.5 | WRBT | 0.215/0.328 |
| A/Chicken/Guangdong/15681/2015 | MT561800 | 15681 | h9.4.2.5 | HRBT | 0.239/1.317 |
| A/Chicken/Guangdong/15690/2015 | MT561801 | 15690 | h9.4.2.5 | DRBT | 0.524/2.476 |
| A/Chicken/Guangdong/15176/2015 | MT561790 | 15176 | h9.4.2.5 | DRBT | 0.646/1.258 |
| A/Chicken/Guangdong/15199/2015 | MT561791 | 15199 | h9.4.2.5 | DRBT | 0.78/1.914 |
| A/Chicken/Guangdong/15798/2015 | MT561802 | 15798 | h9.4.2.5 | HRBT | 0.407/1.922 |
| A/Chicken/Guangdong/15830/2015 | MT561804 | 15830 | h9.4.2.5 | HRBT | 0.243/1.338 |
| A/Chicken/Guangdong/15843/2015 | MT561805 | 15843 | h9.4.2.5 | HRBT | 0.32/1.238 |
| A/Chicken/Guangdong/16302/2015 | MT561809 | 16302 | h9.4.2.5 | HRBT | 0.43/1.96 |
| A/Chicken/Guangdong/16405/2016 | MT561810 | 16405 | h9.4.2.5 | DRBT | 0.755/2.858 |
| A/Chicken/Guangdong/16206/2016 | MT561807 | 16206 | h9.4.2.5 | DRBT | 0.733/1.623 |
| A/Chicken/Guangdong/16818/2016 | MT561818 | 16818 | h9.4.2.5 | HRBT | 0.266/0.655 |
| A/Chicken/Guangdong/16863/2016 | MT561820 | 16863 | h9.4.2.5 | HRBT | 0.477/3.658 |
| A/Chicken/Guangdong/16061/2016 | MT561821 | 16938 | h9.4.2.5 | DRBT | 0.553/1.312 |
| A/Chicken/Guangdong/16240/2016 | MT561806 | 16061 | h9.4.2.5 | DRBT | 0.495/0.823 |
| A/Chicken/Guangdong/16723/2016 | MT561808 | 16240 | h9.4.2.5 | DRBT | 0.623/1.848 |
| A/Chicken/Guangdong/16733/2016 | MT561815 | 16723 | h9.4.2.5 | WRBT | 0.442/0.149 |
| A/Chicken/Guangdong/16733/2016 | MT561816 | 16733 | h9.4.2.5 | HRBT | 0.387/2.628 |
| A/Chicken/Guangdong/16A46/2016 | MT561823 | 16A46 | h9.4.2.5 | HRBT | 0.195/1.096 |
| A/Chicken/Guangdong/3-7/2016 | MT561845 | 3-7 | h9.4.2.5 | HRBT | 0.284/0.902 |
| A/Chicken/Guangdong/16506/2016 | MT561811 | 16506 | h9.4.2.5 | HRBT | 0.358/1.401 |
| A/Chicken/Guangdong/16544/2016 | MT561812 | 16544 | h9.4.2.5 | HRBT | 0.403/1.831 |
| A/Chicken/Guangdong/16770/2016 | MT561817 | 16770 | h9.4.2.5 | HRBT | 0.332/1.133 |
| A/Chicken/Guangdong/16850/2016 | MT561819 | 16850 | h9.4.2.5 | DRBT | 0.511/1.801 |
| A/Chicken/Guangdong/16A24/2016 | MT561822 | 16A24 | h9.4.2.5 | HRBT | 0.318/2.478 |
| A/Chicken/Guangdong/ZG-3/2016 | MT561865 | ZG-3 | h9.4.2.5 | HRBT | 0.27/2.607 |
| A/Chicken/Guangdong/17233/2017 | MT561826 | 17233 | h9.4.2.5 | DRBT | 0.802/2.922 |
| A/Chicken/Guangdong/17355/2017 | MT561829 | 17355 | h9.4.2.5 | DRBT | 0.626/1.669 |
| A/Chicken/Guangdong/17362/2017 | MT561830 | 17362 | h9.4.2.5 | DRBT | 0.186/3.33 |
| A/Chicken/Guangdong/17642/2017 | MT561834 | 17642 | h9.4.2.5 | HRBT | 0.117/1.659 |
| A/Chicken/Guangdong/17237/2017 | MT561827 | 17237 | h9.4.2.5 | WRBT | 0.236/0.269 |
| A/Chicken/Guangdong/17384/2017 | MT561831 | 17384 | h9.4.2.5 | DRBT | 0.636/2.612 |
| A/Chicken/Guangdong/17617/2017 | MT561833 | 17617 | h9.4.2.5 | HRBT | 0.454/0.63 |
| A/Chicken/Guangdong/17699/2017 | MT561836 | 17699 | h9.4.2.5 | DRBT | 0.379/0.83 |
| A/Chicken/Guangdong/17222/2017 | MT561824 | 17222 | h9.4.2.5 | HRBT | 0.269/0.627 |
| A/Chicken/Guangdong/17227/2017 | MT561825 | 17227 | h9.4.2.5 | DRBT | 0.751/1.229 |
| A/Chicken/Guangdong/17263/2017 | MT561828 | 17263 | h9.4.2.5 | HRBT | 0.448/1.24 |
| A/Chicken/Guangdong/17549/2017 | MT561832 | 17549 | h9.4.2.5 | WRBT | 0.12/0.107 |
| A/Chicken/Guangdong/17699/2017 | MT561835 | 17675 | h9.4.2.5 | DRBT | 0.635/1.703 |
| A/Chicken/Guangdong/17728/2017 | MT561837 | 17728 | h9.4.2.5 | HRBT | 0.371/1.95 |
