# Supplementary figures and images for: Variation and Molecular Basis for Enhancement of Receptor Binding of H9N2 Avian Influenza Viruses in China Isolates
Source: Front Microbiol. 2020 Dec 17;11:602124. doi: 10.3389/fmicb.2020.602124 (PMC7773702; doi:10.3389/fmicb.2020.602124)

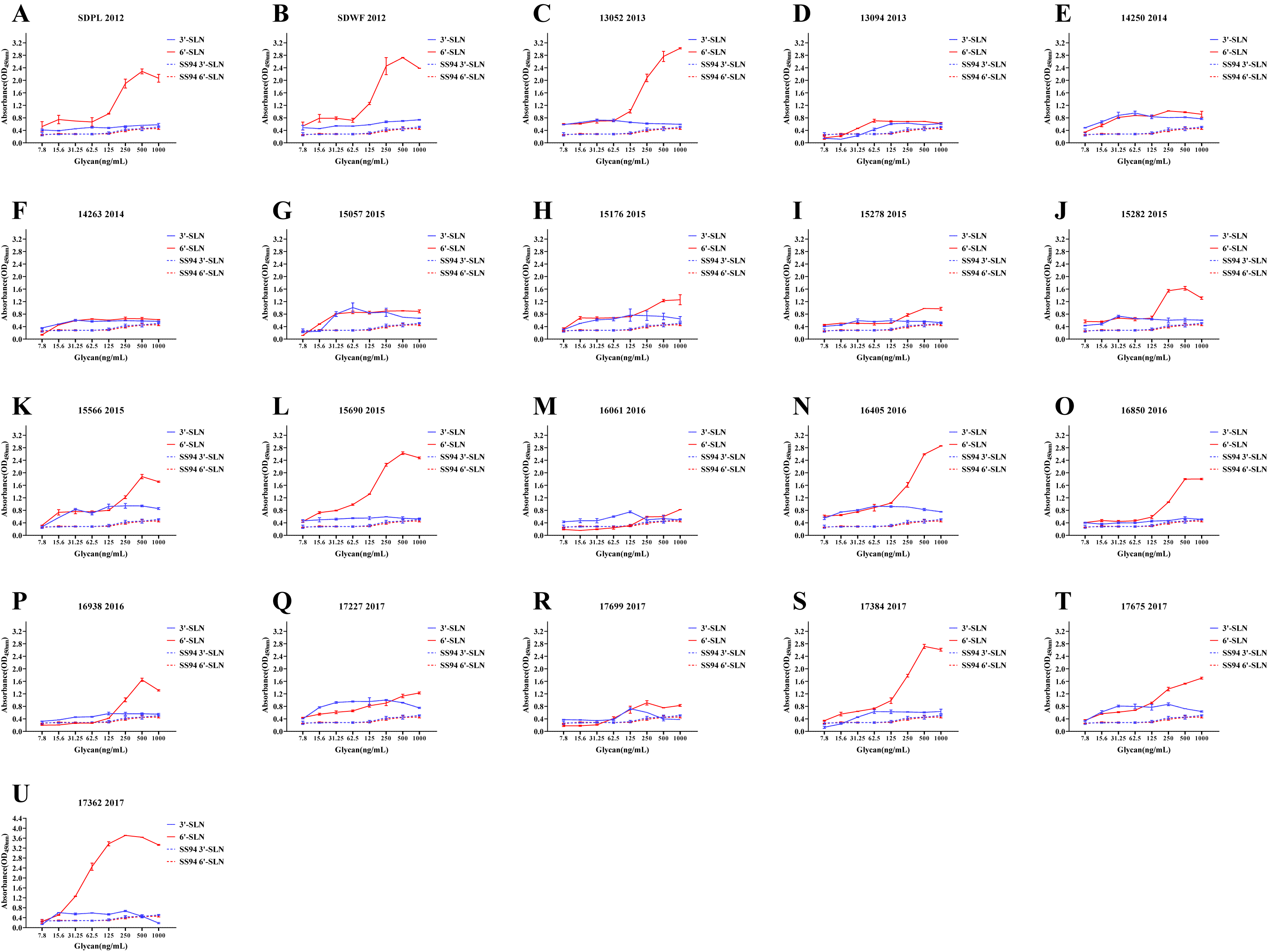

Supplement: Supplementary file 2 [file Image_1.TIF]

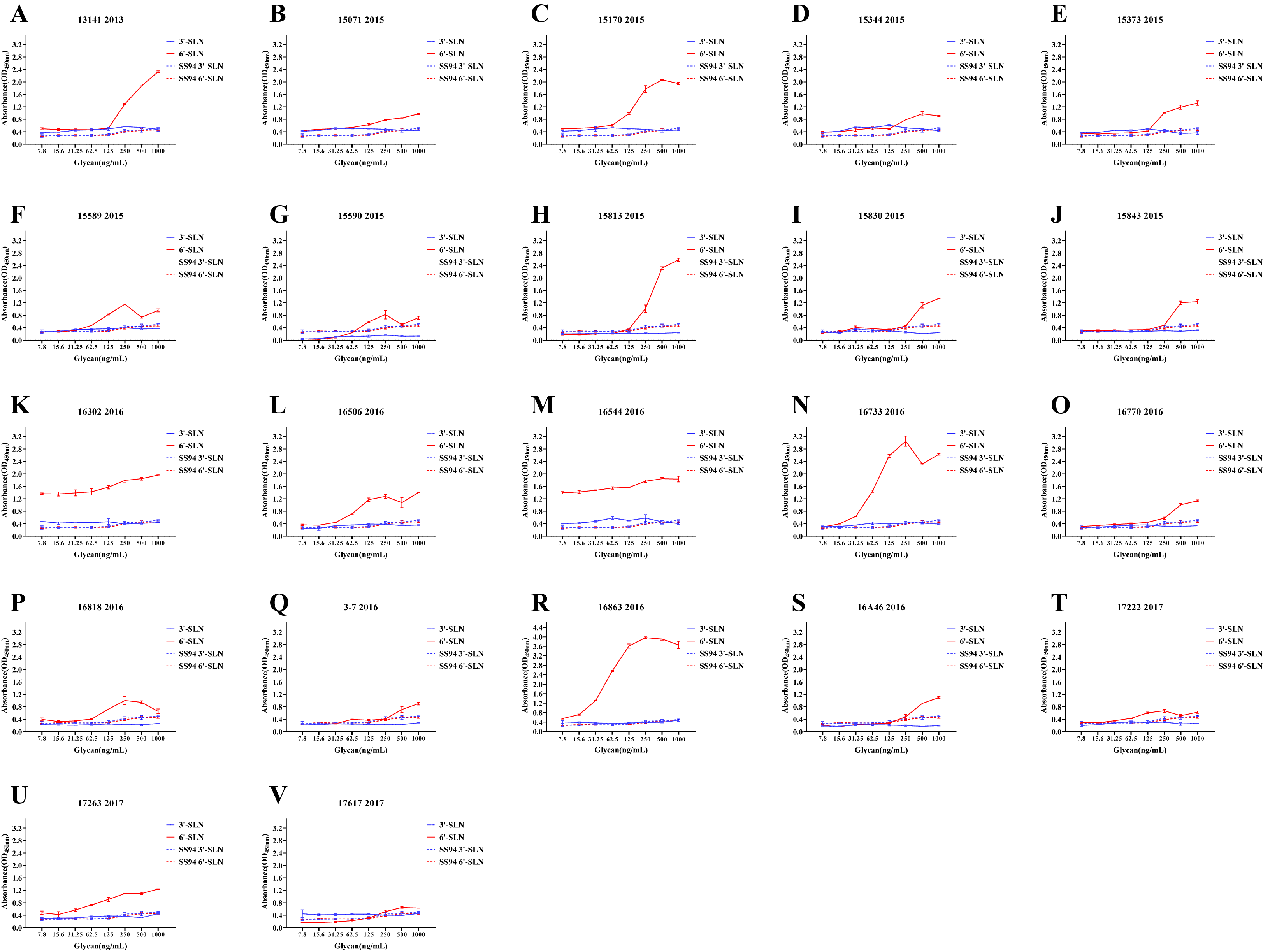

Supplement: Supplementary file 3 [file Image_2.TIF]
